# Supplementary material for: Splice-Junction-Based Mapping of Alternative Isoforms in the Human Proteome
Source: Cell Rep. Author manuscript; Available in PMC 2020 Jan 15. (PMC6961840; doi:10.1016/j.celrep.2019.11.026)
Supplement: 2 [file NIHMS1546469-supplement-2.zip › DF1/PXD006675/AorticValve/AorticValve_1_DPP7_LPGALLPAASGPLQLR.pdf]

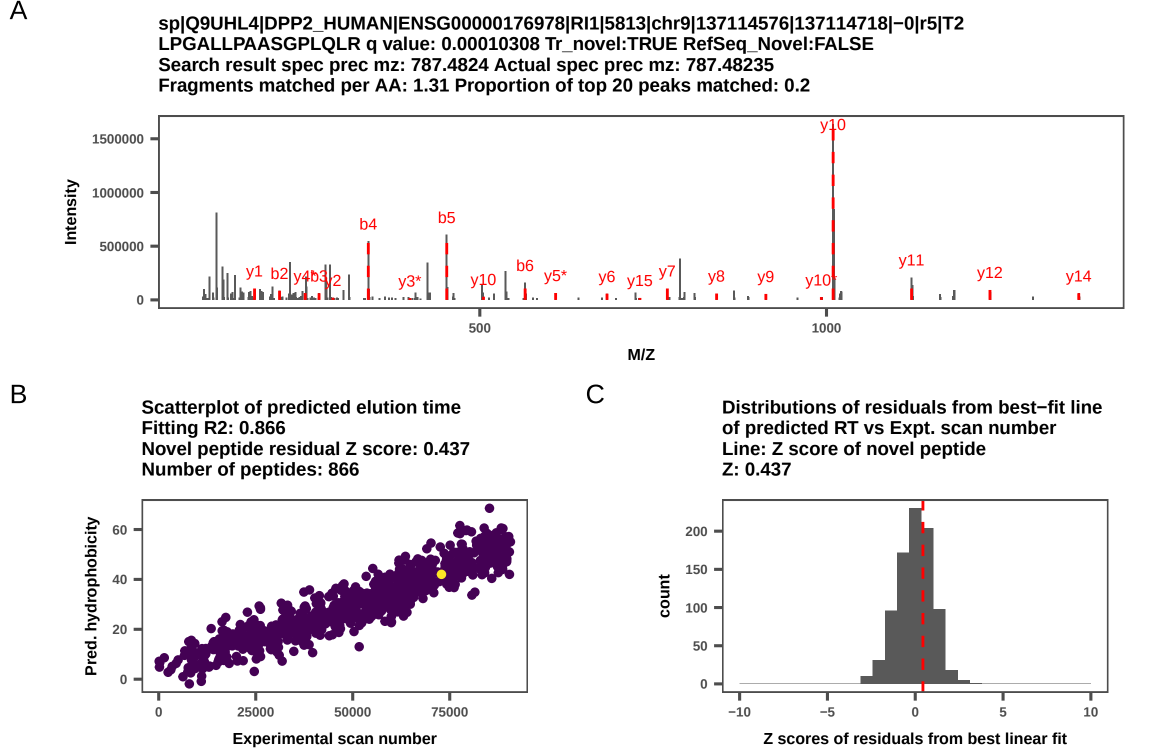
A
sp|Q9UHL4|DPP2_HUMAN|ENSG00000176978|RI1|5813|chr9|137114576|137114718|−0|r5|T2
LPGALLPAASGPLQLR q value: 0.00010308 Tr_novel:TRUE RefSeq_Novel:FALSE
Search result spec prec mz: 787.4824 Actual spec prec mz: 787.48235
Fragments matched per AA: 1.31 Proportion of top 20 peaks matched: 0.2
Intensity
1500000
1000000
500000
0
y1
b2
y4*
b3
y2
b4
y3*
b5
y10
b6
y5*
y6
y15
y7
y8
y9
y10*
y10
y11
y12
y14
500
1000
M/Z
B
Scatterplot of predicted elution time
Fitting R2: 0.866
Novel peptide residual Z score: 0.437
Number of peptides: 866
Pred. hydrophobicity
60
40
20
0
0
25000
50000
75000
Experimental scan number
C
Distributions of residuals from best−fit line
of predicted RT vs Expt. scan number
Line: Z score of novel peptide
Z: 0.437
count
200
150
100
50
0
−10
−5
0
5
10
Z scores of residuals from best linear fit
